# Experiences and Comfort of Young Cancer Patients Discussing Cannabis with Their Providers: Insights from a Survey at an NCI-Designated Cancer Center

**DOI:** 10.1007/s13187-024-02507-9

**Published:** 2024-09-19

**Authors:** Amrit Baral, Bria-Necole A. Diggs, Anurag Aka, Renessa Williams, Nicholas Hernandez Ortega, Ranya Marrakchi El Fellah, Jessica Y. Islam, Marlene Camacho-Rivera, Frank J. Penedo, Denise C. Vidot

**Affiliations:** 1 Division of Epidemiology, Department of Public Health Sciences, University of Miami Miller School of Medicine, 1120 NW 14 Street, Miami, FL 33136 USA; 2 Sylvester Comprehensive Cancer Center, Miami, FL USA; 3 School of Nursing and Health Studies, University of Miami, Coral Gables, FL USA; 4 H. Lee Moffitt Cancer Center and Research Institute, Tampa, FL USA; 5 SUNY Downstate Medical Center, Brooklyn, NY USA

**Keywords:** Cancer, Cannabis, Marijuana, Sociodemographic, Provider, Communication, Treatment

## Abstract

Cannabis use among cancer patients for managing treatment-related symptoms is increasing, yet little is known about patterns in patient-provider communication. This study examines demographic differences in cannabis use communication at a National Cancer Institute-designated cancer center. The analysis included cancer patients aged ≥ 18 years who self-reported current cannabis use (past 30 days) and had visited Sylvester Comprehensive Cancer Center within the past 5 years (*N* = 226). Data were collected via an anonymous electronic survey on REDCap. Responses on patients’ disclosure of cannabis use to cancer doctor/care team and their comfort in discussing cannabis were analyzed. Chi-squared/Fisher’s exact tests and *t*-tests were applied. Logistic regression estimated the associations between age and stage of cancer treatment with patients’ comfort in discussing cannabis use with cancer doctor (oncologist). The sample was 51.8% male and 39.4% Hispanic (mean age, 45.9 years (SD = 15.1)); 41.1% were aged 20–39 years, 43.8% were undergoing treatment, and 35.4% were in follow-up/had finished treatment. Over half (50.4%) did not disclose cannabis use to their cancer doctor/care team. Non-disclosers were more often younger (20–39 years) than disclosers (52.6% vs. 29.5%, *p* < 0.01). Most patients (72.5%) felt comfortable discussing cannabis use with their oncologist; however, younger patients (20–39 years) were more often uncomfortable (40.8%). Logistic regression showed newly diagnosed patients had lower odds (aOR, 0.41; 95% CI, 0.12–0.98) of comfort discussing cannabis compared to those in follow-up/finished treatment. Younger patients (20–39 years) also had lower odds (aOR, 0.11; 95% CI, 0.03–0.40) of feeling comfortable discussing cannabis compared to older patients (≥ 60 years). Age and treatment stage significantly impact the cannabis use disclosure and comfort in discussing it with cancer doctor/care team. These findings underscore the importance of considering age-related factors and treatment status when addressing cannabis use discussions within oncology setting.

## Introduction

The therapeutic landscape of oncology is rapidly evolving with the development and implementation of new treatment modalities and the integration of complementary and alternative medicine aimed at enhancing the quality of life of cancer patients by alleviating cancer symptoms and treatment side effects [1–3]. Cannabis has emerged as a prominent adjunct within complementary and alternative medicine, garnering increasing attention for its potential to manage symptoms such as pain, nausea, mental health symptoms, and appetite loss associated with cancer treatments [4–7]. National estimates indicate that 7–10% of individuals with a history of cancer use cannabis [8]. Single-institution studies suggest a higher prevalence of cannabis use, between 8 and 25%, among those undergoing active cancer treatment [9]. This suggests higher cannabis use among cancer patients in treatment, but the lack of representative studies leaves these estimated differences unexplained. Nonetheless, with the rapidly changing legal landscape around medical and nonmedical cannabis coupled with the diverse availability of products, formulations, and methods of consumption, cannabis use is expected to rise in the United States (US), especially in the context of cancer-related palliative care [10]. Despite its increasing use, patient-provider communication about cannabis in an oncology setting faces significant challenges where the disclosure of cannabis use to oncologists remains a critical yet underexplored area of cancer care.

Effective communication is essential for the safe and beneficial use of cannabis in cancer care, yet research shows both patients and providers often feel uncomfortable discussing it, potentially hindering optimal care and outcomes [11–13]. The stigma surrounding cannabis use, along with legal and regulatory complexities, often obstructs open dialogue between patients and healthcare providers. Many patients remain hesitant to discuss their cannabis use with oncologists due to fears of judgment, stigma, or legal repercussions, which can create barriers to effective communication and comprehensive care [14, 15]. Furthermore, this communication gap results from providers’ lack of knowledge and training about cannabis as well as the absence of standardized guidelines for proper discussions in clinical practice [16]. Existing literature also highlights the significance of patient-provider communication in cancer care, indicating that open discussions can improve symptom management, treatment adherence, and patient satisfaction [17–22].

Prior studies have reported sociodemographic disparities in patient-provider communication about cannabis. For instance, research indicates that men are more likely than women to disclose physician support for medical cannabis use, and younger patients often experience more difficulty in communicating with their healthcare providers [23–26]. Additionally, overall communication between physicians and adult cancer survivors tends to be poorer among Hispanic and Asian patients compared to white, non-Hispanic patients [27–29]. Despite these findings, there is a notable lack of research on this topic focusing specifically on oncology settings. The existing literature does not adequately address how sociodemographic factors influence the disclosure of cannabis use and the comfort level of discussing cannabis with providers in cancer care. Given the increasing normalization and use of cannabis among cancer patients, especially in light of recent legalization trends, understanding these dynamics is crucial for improving patient-provider interactions in oncology.

By analyzing data from patients at a National Cancer Institute (NCI)-designated cancer center, we sought to identify factors that influence patients’ comfort in disclosing cannabis use to their cancer doctor/care team. This research particularly focuses on age-related disparities, as patients of different ages may face unique barriers in disclosing and discussing cannabis with their providers. The primary objectives of this study are threefold. First, it aims to assess the prevalence of cannabis use disclosure among cancer patients and their comfort in discussing cannabis with their oncologist. Second, it seeks to identify sociodemographic and treatment-related factors associated with both disclosure and comfort levels. Third, the study explores how these factors influence patient-provider communication regarding cannabis use. By achieving these objectives, the research seeks to enhance understanding of the dynamics between patients and oncologists concerning cannabis use, ultimately informing strategies to improve patient-provider communication and optimize care outcomes.

## Methods and Materials

### Data Source and Study Sample

Data for this analysis were derived from phase 1 of an ongoing two-phase cross-sectional study, designed to investigate patterns, reasons, and sources of cannabis use among cancer patients. This study was conducted at the Sylvester Comprehensive Cancer Center (SCCC) at the University of Miami Miller School of Medicine, Miami, FL, utilizing a harmonized questionnaire developed in collaboration with eleven NCI-designated cancer centers (see measure: https://epi.grants.cancer.gov/clinical/nci-cannabis-supplement-core-measures-questionnaire.pdf). Cancer patients aged 18 or older, seen at Sylvester Comprehensive Cancer Center (SCCC) within the last 5 years of cancer treatment, including surgery, radiation, chemotherapy, immunotherapy, and follow-up, were eligible. Participants, either in active treatment or within 5 years of initial treatment, confirmed their eligibility via a REDCap question before starting the survey. Recruitment occurred through phone calls, electronic health portals (e.g., MyUHealthChart), flyers in clinic waiting rooms, and direct contact with care teams. The study, approved by the University of Miami Institutional Review Board and SCCC’s Protocol Review and Monitoring Committee, obtained informed consent and collected anonymous data via REDCap from October 2021 to June 2023. The overall study sample consisted of 416 adults.

Included in this analytic sample were cancer patients (*N* = 226) who self-reported current cannabis use (past 30 days), disclosure of cannabis use, and their comfort in discussing cannabis with their cancer doctor and cancer care team.

### Study Measures

The primary variables of interest were the disclosure of cannabis use by cancer patients and their comfort level in discussing cannabis with their cancer doctor and cancer care team. For the measure of disclosure, current cannabis users were asked, “Does your cancer doctor know you are using cannabis?” with the following response options: (1) *None of my healthcare providers know*, (2) *I have not told my cancer doctor/team, but another healthcare provider knows*, and (3) *My cancer doctor/team knows*. This variable was recoded into a binary yes/no variable for analysis where yes represented participants that communicated with their cancer doctor/team. Additionally, current cannabis users were asked, “Do you feel comfortable discussing cannabis with your cancer doctor?” with response options of yes or no.

The independent variables of interest included sociodemographic characteristics and the stage of cancer treatment. Sociodemographic variables including age, sex at birth, race/ethnicity, income, employment status, education level, marital status, health care coverage, sexual orientation, and country of birth were collected through self-report. The stage of cancer treatment was also self-reported, with participants being asked, “Where are you in your cancer treatment?” The response options were as follows: (1) *Newly diagnosed*, (2) *Currently undergoing treatment*, (3) *Finished therapy/follow-up*, and (4) *Not receiving treatment*.

### Statistical Analysis

Descriptive statistics were employed to summarize the sociodemographic characteristics of cancer patients overall and by their self-reported disclosure of cannabis use to cancer doctor/cancer care team (Table 1). Chi-square tests or Fisher’s exact tests, as appropriate, were used to compare proportions. Similarly, sociodemographic characteristics were summarized by patients’ comfort level in discussing cannabis with their cancer doctor (oncologist) (Table 2). For comparisons of means, *t*-tests were utilized. A multivariable logistic regression analysis was performed to estimate the association between age and stage of cancer treatment with patients’ comfort in discussing cannabis use with their cancer doctor/cancer care team. Adjusted odds ratios and 95% confidence intervals are reported (Table 3). All statistical analyses were conducted using SAS Analytics 9.4, with a two-tailed alpha level set at 0.05.

**Table 1:** Sociodemographic characteristics of cancer patients by disclosure of cannabis use to their oncologists (*N* = 226, current cannabis users)

**Characteristics**	**Overall** (*N* = 226)	**Does your cancer doctor know you are using cannabis?**		*p*-value
		**Yes** (*n* = 112, 49.6%)	**No** (*n* = 114, 50.4%)	
**Age (yrs.)**				< .0001
*Mean (SD)*	45.9 (15.1)	50.1 (14.5)	41.7 (14.6)	
**Age categories (yrs.)**				
20–39	93 (41.1)	33 (29.5)	60 (52.6)	< .01
40–59	79 (35.0)	44 (39.3)	35 (30.7)	
≥ 60	54 (23.9)	35(31.2)	19 (16.7)	
**Sex at birth**				0.79
Male	117 (51.8)	57 (50.9)	60 (52.6)	
Female	109 (48.2)	55 (49.1)	54 (47.4)	
**Ethnicity**				0.26
Hispanic	89 (39.4)	40 (35.7)	49 (43.0)	
Non-Hispanic	137 (60.6)	72 (64.3)	65 (57.0)	
**LQBTQ**				0.41
No	202 (89.4)	102 (91.1)	100 (87.7)	
Yes	24 (10.6)	10 (8.9)	14 (12.3)	
**Education**				0.01
High school or less	32 (14.2)	14 (12.5)	18 (15.8)	
Some college/technical	77 (34.1)	28 (25.1)	49 (43.0)	
College graduate	57 (25.2)	35 (31.2)	22 (19.3)	
Postgraduate	60 (26.5)	35 (31.2)	25 (21.9)	
**Employment status**				< .01
Employed	117 (51.8)	59 (52.7)	58 (50.9)	
Unemployed	44 (19.5)	13 (11.6)	31 (27.2)	
Other	65 (28.7)	40 (35.7)	25 (21.9)	
**Income (in US$)**				0.08
0–34,999	54 (23.9)	25 (22.3)	29 (25.4)	
35,000–74,999	65 (28.8)	26 (23.2)	39 (34.2)	
≥ 75,000	107 (47.3)	61 (54.5)	46 (40.3)	
**Marital status**				0.05
Married/partnered	155 (68.6)	70 (62.5)	85 (74.6)	
Unmarried	71 (31.4)	42 (37.5)	29 (25.4)	
**Nativity (US-born)**				0.50
Yes	172 (76.4)	87 (78.4)	85 (74.6)	
No	53 (23.6)	24 (21.6)	29 (25.4)	
**Healthcare coverage**				< .0001
Yes	188 (83.2)	108 (96.4)	80 (70.2)	
No	38 (16.8)	4 (3.6)	34 (29.8)

**Table 2:** Sociodemographic characteristics of cancer patients by their comfort of discussing cannabis with their oncologists (*N* = 211, current cannabis users)

Characteristics	Do you feel comfortable discussing cannabis with your cancer doctor? (*N* = 211)		*p*-value
	**Yes** (*n* = 153, 72.5%)	**No** (*n* = 58, 27.5%)	
**Age (yrs.)**			< .0001
*Mean (SD)*	49.2 (15.0)	36.7 (10.9)	
**Age categories (yrs.)**			
20–39	50 (32.7)	38 (65.5)	< .0001
40–59	56 (36.6)	17 (29.3)	
≥ 60	47 (30.7)	3 (5.2)	
**Sex at birth**			0.87
Male	81 (52.9)	30 (51.7)	
Female	72 (47.1)	28 (48.3)	
**Ethnicity**			0.27
Hispanic	56 (36.6)	26 (44.8)	
Non-Hispanic	97 (63.4)	32 (55.2)	
**LQBTQ**			0.41
No	138 (90.2)	50 (86.2)	
Yes	15 (9.8)	8 (13.8)	
**Education**			0.04
High school or less	21 (13.7)	10 (17.2)	
Some college/technical	46 (30.1)	27 (46.6)	
College graduate	41 (26.8)	13 (22.4)	
Postgraduate	45 (29.4)	8 (13.8)	
**Employment status**			< .01
Employed	81 (53.0)	28 (48.3)	
Unemployed	23 (15.0)	19 (32.7)	
Other	49 (32.0)	11 (19.0)	
**Income (in US$)**			0.37
0–34,999	37 (24.2)	15 (25.9)	
35,000–74,999	40 (26.1)	20 (34.5)	
≥ 75,000	76 (49.7)	23 (39.6)	
**Marital status**			0.10
Married/partnered	101 (66.0)	45 (77.6)	
Unmarried	52 (34.0)	13 (22.4)	
**Nativity (US-born)**			0.97
Yes	115 (75.7)	44 (75.9)	
No	37 (24.3)	14 (24.1)	
**Healthcare coverage**			< .001
Yes	135 (88.2)	39 (67.2)	
No	18 (11.8)	19 (32.8)	

Note: There were 15 missing responses on the question: “Do you feel comfortable discussing cannabis with your cancer doctor?”

**Table 3:** Multivariable logistic regression analysis with odds of patient comfort in discussing cannabis use with oncologists (*N* = 211, current cannabis users)

Variables	Adjusted odds ratio (95% confidence interval)
**Age in years (reference ≥ 60)**	
20–39	0.11 (0.03–0.40)
40–59	0.23 (0.10–0.89)
**Sex at birth (reference = female)**	
Male	0.92 (0.47–1.80)
**Cancer treatment stage (reference = finished therapy/follow-up)**	
Newly diagnosed	0.41 (0.12–0.98)
Currently under treatment	1.53 (0.71–3.30)
Not receiving treatment	2.31 (0.24–22.0)

## Results

Table 1 presents the sociodemographic characteristics of 226 cancer patients based on whether they disclosed their cannabis use to their cancer doctor/cancer care team. Of the patients, 49.6% (*n* = 112) disclosed their use, while 50.4% (*n* = 114) did not. The mean age of the sample was 45.9 years (SD = 15.1). Disclosers were significantly older, with a mean age of 50.1 years (SD = 14.5), compared to non-disclosers (mean age = 41.7 years, SD = 14.6, *p* < 0.0001). Age categories also showed a significant difference (*p* < 0.01). Among disclosers, 29.5% were aged 20–39 years, compared to 52.6% of non-disclosers (*p* < 0.01). Conversely, 31.2% of disclosers were aged 60 + years, while only 16.7% of non-disclosers were in this category. There were no significant differences in sex at birth (*p* = 0.79). The proportion of Hispanic patients was similar between groups, with 35.7% of disclosers and 43.0% of non-disclosers (*p* = 0.26). Non-Hispanic patients comprised 64.3% of disclosers and 57.0% of non-disclosers. The difference based on LGBTQ status was not statistically significant (*p* = 0.41).

Education level was associated with disclosure. Among disclosers, 31.2% had a college degree, compared to 19.3% of non-disclosers (*p* = 0.01). Conversely, 43.0% of non-disclosers had some college or technical education, while only 25.1% of disclosers did. Employment status showed a significant association with disclosure (*p* < 0.01). Among unemployed respondents, 70.5% did not disclose cannabis use compared to 29.5% that did disclose. Among employed respondents, 50.4% disclosed cannabis use and 49.6% did not disclose cannabis use. Income levels did not statistically differ significantly between the groups (*p* = 0.08), although a higher percentage of disclosers had an income of $75,000 + (54.5%) compared to non-disclosers (40.3%). There were no significant differences in disclosure based on marital status (*p* = 0.05) and nativity (*p* = 0.50). Healthcare coverage was significantly associated with disclosure. A high percentage of patients with healthcare coverage disclosed their cannabis use (96.4%) compared to those without coverage (70.2%) (*p* < 0.0001).

**Fig. 1:**
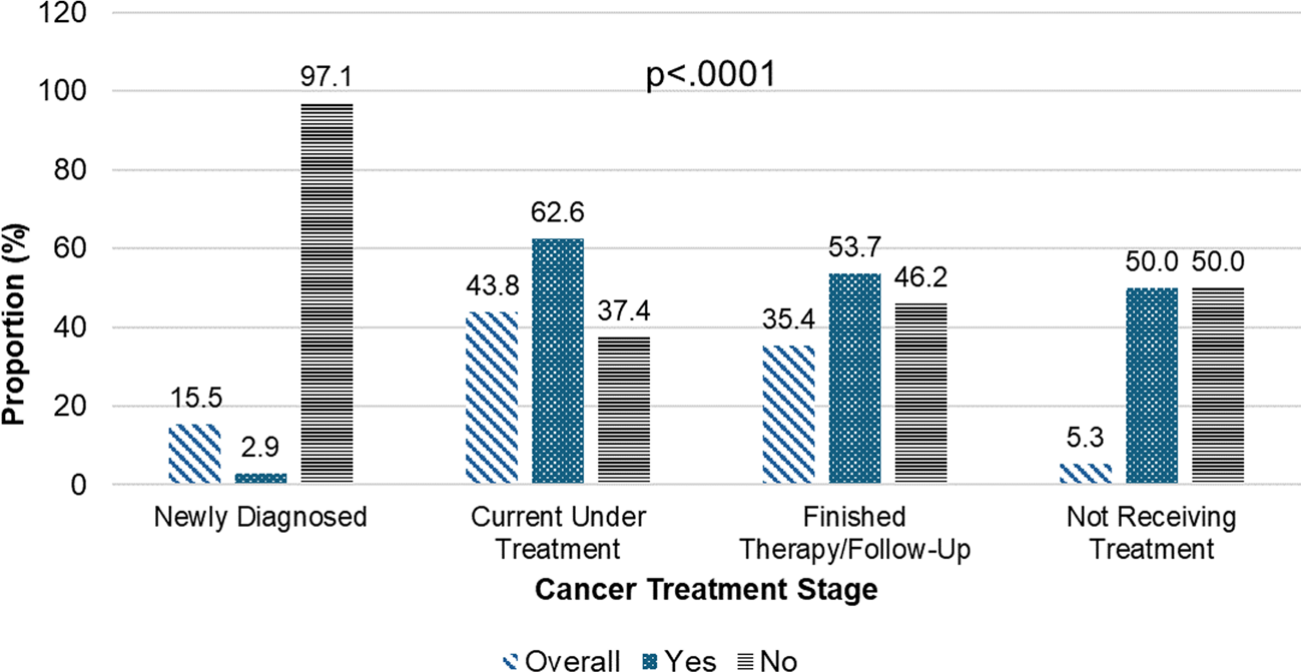
Disclosure of cannabis use to oncologists by cancer treatment stage among self-reporting cancer patients (*N* = 226, current cannabis users)

Figure 1 illustrates disclosure rates of cannabis use among cancer patients at different treatment stages. Significant variations were observed (*p* < 0.0001). Only 2.9% of newly diagnosed patients disclosed their cannabis use, with 97.1% not disclosing. Newly diagnosed patients made up 15.5% of the sample. Among those currently undergoing treatment, 62.6% disclosed, while 37.4% did not, constituting 43.8% of the sample. For patients who completed therapy or were in follow-up, 53.7% disclosed, and 46.2% did not, making up 35.4% of the sample. Among those not receiving treatment, 50.0% disclosed and 50.0% did not, representing 5.3% of the sample.

Table 2 presents the sociodemographic characteristics of cancer patients based on their comfort in discussing cannabis use with their oncologist. The analysis included 211 current cannabis users: 72.5% were comfortable discussing cannabis, while 27.5% were not. The mean age of patients comfortable discussing cannabis was 49.2 years (SD = 15.0), significantly older than the 36.7 years (SD = 10.9) for those uncomfortable (*p* < 0.0001). Among comfortable patients, 32.7% were aged 20–39 years, compared to 65.5% of those uncomfortable (*p* < 0.0001). Conversely, 5.2% of uncomfortable patients were aged 60 years or older, while 30.7% of comfortable patients were in this age category. There was no significant difference in comfort levels based on sex at birth (*p* = 0.87).

The proportion of Hispanic patients was slightly higher among those uncomfortable discussing cannabis (44.8%) compared to those comfortable (36.6%), but this difference was not significant (*p* = 0.27). LGBTQ status did not significantly affect comfort (*p* = 0.41). Educational attainment was significantly associated with comfort. Among those comfortable, 13.7% had a high school education or less, versus 17.2% of those uncomfortable. In contrast, 46.6% of uncomfortable patients had some college or technical education, compared to 30.1% of those comfortable (*p* = 0.04). College graduates were 26.8% of those comfortable and 22.4% of those uncomfortable, while 29.4% of comfortable patients had postgraduate education versus 13.8% of uncomfortable patients.

Employment status was also significantly related to comfort (*p* < 0.01) Unemployed participants were more likely to feel uncomfortable (32.7%) compared to those who felt comfortable (15.0%). Income levels (*p* = 0.37), nativity (*p* = 0.97), and marital status (*p* = 0.1) did not show a significant association with comfort. Whereas healthcare coverage was significantly associated with comfort: 88.2% of patients with coverage felt comfortable discussing cannabis, compared to 67.2% of those without (*p* < 0.001).

**Fig. 2:**
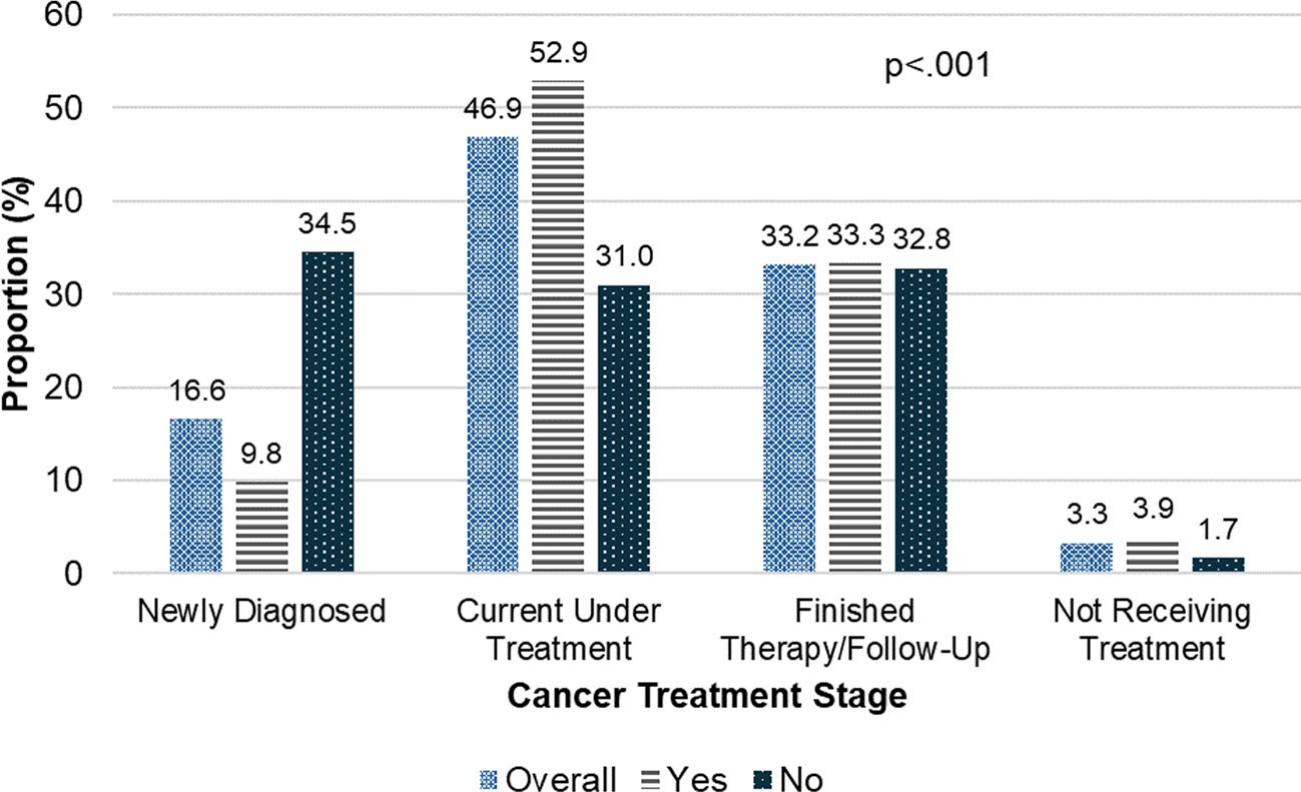
Patient comfort in discussing cannabis use with oncologists by cancer treatment stage (*N* = 211, current cannabis users)

Figure 2 illustrates the proportion of cancer patients who feel comfortable discussing their cannabis use with their oncologist, stratified by cancer treatment stage. Among the overall sample (*N* = 221), 16.6% were newly diagnosed, 46.9% were currently under treatment, 33.2% had finished therapy and were in follow-up, and 3.3% were not receiving treatment. Patients who were newly diagnosed and felt comfortable discussing cannabis use with their oncologists comprised 9.8%, compared to 34.5% who did not feel comfortable. Among those currently under treatment, 31.0% felt comfortable discussing cannabis use, while 52.9% did not. For patients who had finished therapy and were in follow-up, 33.3% felt comfortable, nearly equal to the 32.8% who did not feel comfortable. Finally, among patients not receiving treatment, 1.7% felt comfortable discussing cannabis use with their oncologists, compared to 3.9% who did not. The differences in comfort levels across the cancer treatment stages were statistically significant (*p* < 0.001).

The multivariable logistic regression analysis revealed several significant factors associated with patient comfort in discussing cannabis use with oncologist (Table 3). Age was a significant factor in patient comfort in discussing cannabis with their cancer doctor. Patients aged 20–39 years had significantly lower odds of feeling comfortable discussing cannabis use with their oncologists compared to those aged 60 years or older (aOR = 0.11; 95% CI, 0.03–0.40). Similarly, patients aged 40–59 years also had lower odds of comfort compared to the reference group (aOR = 0.23; 95% CI, 0.10–0.89). The analysis showed that sex at birth was not a significant predictor of comfort levels. Males had an aOR of 0.92 (95% CI, 0.47–1.80) compared to females, indicating no significant difference in comfort levels based on gender. The stage of cancer treatment was significantly associated with patient comfort in discussing cannabis use. Patients who were newly diagnosed with cancer had significantly lower odds of feeling comfortable discussing cannabis use compared to those who had finished therapy or were in follow-up (aOR = 0.41; 95% CI, 0.12–0.98). Conversely, patients currently under treatment showed higher, but not statistically significant, odds of comfort (aOR = 1.53; 95% CI, 0.71–3.30). Patients not receiving treatment had the highest odds of comfort (aOR = 2.31; 95% CI, 0.24–22.0), though this result was not statistically significant.

## Discussion

This study aimed to examine the disclosure of cannabis use and the comfort of cancer patients in discussing cannabis with their oncologists. We found that 49.6% of cancer patients disclosed their cannabis use to their cancer doctor/care team, while a majority (50.4%) did not. Key findings indicate that older patients were more likely to disclose their cannabis use compared to younger patients, and newly diagnosed patients were less likely to disclose cannabis use than those currently undergoing treatment or in follow-up stages. Additionally, comfort in discussing cannabis with oncologists was higher among older patients and those currently undergoing treatment. Sociodemographic factors, such as education level and unemployment, were significantly associated with both disclosure and comfort levels.

Our findings align with previous studies that have highlighted the complexities of patient-provider communication regarding cannabis use in cancer care. Similar to our results, a study found that older adult cannabis users discuss their cannabis use with healthcare providers more often than younger users [30]. However, there is no evidence as of now for a direct comparison of our findings in the setting of oncology. Possible reasons for older patients being more likely to discuss cannabis with their providers and open to discussing include longer history of managing their disease and treatments, and greater confidence in their healthcare relationships with physicians and the healthcare system [11, 31, 32]. A study conducted by Turner et al. (2023) to describe consumption of cannabis in young adults (18–39 years) found that while most young adults felt comfortable discussing cannabis with providers, overall patient-provider communication about medical cannabis remains limited [33]. Our observation that patients currently undergoing treatment are more likely to disclose cannabis use and feel comfortable discussing it with oncologists/cancer care teams is consistent with findings from another that suggest active treatment phases heighten patient engagement and communication [34]. Potential explanations for these dynamics are likely because patients undergoing treatment longer may have more access to support networks, including other patients who use cannabis, which can provide them with the confidence and knowledge needed to initiate these conversations. Over time, patients develop a trust-based relationship with their healthcare providers, making them feel more comfortable discussing alternative therapies such as cannabis.

On the other hand, newly diagnosed patients often face a barrage of new information and are primarily focused on understanding their diagnosis and initial treatment options [35]. This overwhelming situation leaves little room for exploring alternative treatments like cannabis. Additionally, they might not be aware of the potential benefits or risks associated with cannabis use, making them less likely to bring it up in conversations with their healthcare providers [36]. Many newly diagnosed patients are unsure whether their oncologists or healthcare providers would endorse or support the use of cannabis [37, 38]. Evidence shows that even among patients already using cannabis, discussions about it are often patient-initiated, indicating a lack of proactive communication from healthcare providers [39].

Furthermore, our findings indicated that patients with higher educational attainment were more likely to disclose their cannabis use and feel comfortable discussing it with their oncologists or cancer care team. Conversely, unemployed patients were less likely to disclose their cannabis use and feel comfortable having such discussions. As of now, there are no studies for direct comparisons of our findings in the context of cancer care. However, evidence shows that patients in general with higher education levels and those who were employed were more inclined to report their cannabis use to healthcare providers [40]. This openness could be attributed to their better understanding of the benefits and risks associated with cannabis and their perception of less stigma surrounding its use. Research indicates that unemployed individuals often hesitate to disclose sensitive health information to healthcare providers due to fear of stigma, judgment, and potential negative consequences [41, 42]. Another study by King et al. (2024) reported that annual household income (*p* = 0.04) had statistically significant associations with the frequency of cannabis use disclosure [14].

This study has several strengths, including the use of an anonymous survey, which likely reduced social desirability bias and encouraged honest reporting and a high participation rate with 70% of participants declining compensation. The comprehensive collection of sociodemographic data allowed for detailed analysis. Additionally, the multivariable logistic regression analysis provided robust results, and the focus on age-related disparities offered valuable insights, especially given the lack of existing evidence on this topic. However, the study’s cross-sectional design limits causal inferences, and reliance on self-reported data introduces potential recall and social desirability biases. The sample size, while adequate, may not capture all nuances of the broader cancer patient population, and the findings may not be generalizable to other clinical settings or geographical locations. However, our findings contribute to the current climate of patient-provider discourses on cannabis in an oncology setting.

### Implications for Clinical and Public Health Practice

Our findings suggest the need for oncologists and cancer care team to proactively address the topic of cannabis use with their patients, particularly with younger and newly diagnosed patients who may be less likely to initiate these conversations. Healthcare providers should be aware of the sociodemographic factors that may influence patients’ willingness to disclose cannabis use and their comfort in discussing it. Tailoring communication strategies to account for these factors can improve patient-provider interactions and ensure that patients receive comprehensive guidance on the use of cannabis in their cancer care. From a public health perspective, our findings underscore the need for educational interventions targeting cancer patients to empower them to discuss cannabis use openly with their healthcare providers. Policy-wise, standardizing guidelines for discussing cannabis use in oncology can alleviate stigma and facilitate more open conversations.

To address the communication gap between newly diagnosed cancer patients and their healthcare providers regarding cannabis use, several strategic interventions can be implemented to enhance patient-provider dialogues and improve overall care:

- Develop accessible educational materials: Creating educational materials that outline key topics for discussion with healthcare providers, including cannabis use, could enhance patient-provider communication. These materials should feature example questions and scenarios to guide patients in preparing for their appointments. Question prompt lists (QPLs) have been proven effective in fostering communication about sensitive topics by encouraging patients to ask more questions and reducing unmet information needs while improving recall without elevating anxiety[43, 44]. Tailoring QPLs to specific cancer types, cultural contexts, and stages of care can further enhance their effectiveness [45, 46].
- Leverage digital platforms: Utilizing online platforms, such as hospital websites, patient portals, and social media, to share information about the importance of discussing cannabis use with providers. Although online platforms can facilitate these discussions, further research is needed to establish evidence-based clinical guidelines for cannabis use in medical practice [36, 47].
- Telehealth and virtual consultations: Encourage the use of telehealth services where patients might feel more comfortable discussing sensitive topics like cannabis use from the privacy of their own homes. Telehealth has also been successfully implemented in integrative oncology consultations, with patients showing increased interest in lifestyle counseling and supplement use [48]. However, some patients perceive in-person visits as providing better communication and overall quality of care [49].
- Promote support groups: Support groups for newly diagnosed cancer patients can serve as valuable resources for sharing experiences and strategies related to discussing cannabis use with providers. Peer support has been highly valued in psychosocial support programs, emphasizing the importance of social interaction and complementary therapies [50]. By facilitating conversations with peers who have successfully navigated these discussions, support groups can empower other patients to engage in similar dialogues with their providers [50].
- Incorporate cannabis discussion into standard care protocols: As cannabis use becomes increasingly prevalent, it is essential to integrate discussions about cannabis into standard care protocols. Providers should routinely inquire about cannabis use during patient intake and engage in open, non-judgmental conversations [51]. However, existing hesitations among providers due to conflicting beliefs and a lack of clear guidelines highlight the need for training in motivational interviewing and person-centered approaches. These skills can help providers discuss the risks and benefits of cannabis more comfortably and reduce stigma [33, 52].
- Training providers on patient engagement: Training programs that focus on patient engagement have successfully improved healthcare providers’ knowledge and attitudes toward medical cannabis [51, 53]. Providers should be trained to engage in open, nonjudgmental conversations about cannabis use, even if they do not prescribe it [36, 37]. Incorporating strategies such as motivational interviewing into medical education can enhance providers’ communication skills, reduce stigma, and improve patient interactions [36].

## Conclusion

In conclusion, our findings highlight the importance of open and initiative-taking communication between cancer patients and their oncologists regarding cannabis use. By understanding the factors that influence disclosure and comfort levels, healthcare providers can better support their patients in making informed decisions about their treatment options. Future research should explore longitudinal trends in cannabis use disclosure and communication comfort, as well as the impact of targeted interventions on improving these aspects of cancer care.

## Data Availability

De-identified data utilized in this study may be shared with investigators upon reasonable request to the principal investigator (Denise C. Vidot, PhD) by e-mail: dvidot@miami.edu.
